# Peripheral blood stem cells *versus* bone marrow graft for non-T-depleted haploidentical transplantation with post-transplant cyclophosphamide in patients with secondary acute myeloid leukemia in first complete remission: A study from the ALWP/EBMT

**DOI:** 10.1038/s41409-026-02823-2

**Published:** 2026-03-25

**Authors:** Arnon Nagler, Ryszard Swoboda, Allain-Thibeault Ferhat, Didier Blaise, Mariya Koc, Anna Maria Raiola, Alessandro Busca, Jiri Pavlu, Stefania Bramanti, Maija Itälä-Remes, Mercedes Colorado, Jan Vydra, Alexander Kulagin, Ali Bazarbachi, Jaime Sanz, Mohamad Mohty, Fabio Ciceri

**Affiliations:** 1 Division of Hematology, Sheba Medical Center, Tel Hashomer, Israel; 2 Maria Sklodowska-Curie National Research Institute of Oncology, Gliwice Branch, Gliwice, Poland; 3 EBMT Paris study office; Department of Haematology, Saint Antoine Hospital; INSERM UMR 938, Sorbonne University, Paris, France; 4 Programme de Transplantation & Therapie Cellulaire, Marseille, France; 5 Medicana International Hospital Istanbul, Istanbul, Turkey; 6 IRCCS Ospedale Policlinico San Martino, Genova, Italy; 7 S.S.C.V.D Trapianto di Cellule Staminali, Torino, Italy; 8 Imperial College Hammersmith London, London, UK; 9 IRCCS Istituto Clinico Humanitas, Rozzano Milano, Italy; 10 Turku University Hospital, Turku, Finland; 11 Hospital U. Marqués de Valdecilla, Santander, Spain; 12 Institute of Hematology and Blood Transfusion, Prague, Czech Republic; 13 RM Gorbacheva Research Institute, Pavlov University, Petersburg, Russian Federation; 14 Hematology-Oncology Division, Department of Internal Medicine, American University of Beirut Medical Center, Beirut, Lebanon; 15 Hematology Department, Hospital Universitari i Politècnic La Fe, Valencia Departament de Medicina Universitat de Valencia, CIBERONC, Instituto Carlos III, Madrid, Spain; 16 Sorbonne University, Department of Haematology, Saint Antoine Hospital; INSERM UMR 938, Paris, France; 17 Ospedale San Raffaele, Haematology and BMT, Milano, Italy

**Keywords:** Diseases, Haematological diseases

## Abstract

Haploidentical stem cell transplantation (haplo-HSCT) with post-transplant cyclophosphamide (PTCy) is a therapeutic option for secondary acute myeloid leukemia (sAML). The study aimed to compare peripheral blood stem cells (PBSC) *vs*. bone marrow (BM) as a graft source for haplo-HSCT with PTCy in patients with sAML in first complete remission. A total of 554 patients were included, BM = 136, PBSC = 418. Median follow-up was 3 years. The median year of transplant was 2018 (range, 2010–2021). The antecedent hematological disease was myelodysplastic syndrome /myeloproliferative neoplasms in most patients. Patients in the BM group were younger, median age 59.2 vs. 61.7 years (*p* = 0.008), and received myeloablative conditioning more frequently (66.4% *vs*. 47.4%, *p* < 0.001). Time from diagnosis to haplo-HSCT was 5.3 *vs*. 4.8 months, respectively (*p* = 0.019). Performance status, cytogenetic risk, gender, cytomegalovirus serostatus, and donor age did not differ. There were no differences between the groups with respect to main transplantation outcomes. In conclusion, outcomes of haplo-HSCT with PTCy in sAML with either PBSC or BM grafts are similar, with no differences in major transplantation outcomes.

## Introduction

Secondary acute myeloid leukemia (sAML) comprises a heterogeneous group of diseases evolving from a pre-existing hematologic disorder, predominantly myelodysplastic syndrome (MDS) and myeloproliferative neoplasm (MPN) or as a complication of prior cytotoxic chemotherapy or radiation therapy [1–8]. Despite new drugs incorporated into the armamentarium for sAML treatment in the last few years [9], allogeneic hematopoietic stem cell transplantation (HSCT) remains the only potentially curable option for patients with sAML [10–13]. However, outcomes of transplants from a human leukocyte antigen (HLA)-matched sibling donor (MSD) and a matched unrelated donor (MUD) in sAML seem to be poorer than in patients with de novo AML [12]. In contrast, haploidentical donor stem cell transplantation (haplo-HSCT) combined with post-transplantation cyclophosphamide (PTCy) has shown promise, with significantly reduced non-relapse mortality (NRM) and incidence of graft-*versus*-host disease (GVHD) [14, 15], but with potentially an enhanced graft-*versus*-leukemia (GVL) effect [16]. Our recent work demonstrated that the results of haplo-HSCT with PTCy in sAML are not significantly different from those in de novo AML, and that haplo-HSCT may rescue 30-60% of patients with sAML, including those with relapsed/refractory disease, suggesting that haplo-HSCT with PTCy may be able to overcome some of the poor prognosis of sAML patients [17, 18]. Although the original haplo-HSCT with PTCy originated with bone marrow (BM) as the source of stem cells [19], more and more haplo-HSCTs are currently performed with mobilized peripheral blood stem cells (PBSC) [20, 21]. Multiple single-institution and registry-based studies have compared PBSC *versus* BM grafts for haplo-HSCT with PTCy in various haematological malignancies, demonstrating somewhat different results but generally a lower incidence of acute (a) and chronic (c) GVHD with BM grafts [21–25], while engraftment was higher in some of the studies with PBSC grafts [23, 24]. In some of the previous studies with PTCy-based haplo-HSCT, especially with reduced intensity conditioning (RIC), relapse incidence (RI) was lower with PBSC *versus* BM grafts [21, 22]. Notably, in our recent study focusing on mostly de novo AML with more than 800 patients (de novo 96%, sAML-4%) over the age of 60 years undergoing haplo-HSCT with PTCy following RIC, we demonstrated a lower risk of relapse and better leukemia-free survival (LFS) in patients receiving PBSC grafts when compared to those transplanted with BM as a graft source [26]. We concluded that PBSC can be an option to overcome the high risk of relapse in elderly de novo AML patients undergoing haplo-HSCT with PTCy with RIC by inducing a stronger GVL effect [26]. However, none of the previous studies that compared PBSC *versus* BM grafts as the stem cell source in haplo-HSCT with PTCy focused specifically on sAML. This assessment is of special clinical importance in sAML, a high risk leukemia with high relapse risk due to multiple factors including the antecedent hematological disorder, older age, more aggressive biology of the leukemia with adverse cytogenetics and poor-risk mutation profile, lower susceptibility and lower ability to tolerate chemotherapy, among others [1, 6–8, 27]. The GVL is probably of crucial significance in this patient category and one can envision, based on our previous results in de novo AML patients ≥ 60 years described above [25] and previous literature, that using PBSC compared to BM grafts for haplo-HSCT with PTCy in sAML will result in lower RI and better transplantation outcomes. We therefore assessed the results of haplo-HSCT with PTCy in sAML patients transplanted in first complete remission (CR1), comparing PBSC or BM as a graft source, taking advantage of the dataset of the Acute Leukemia Working Party (ALWP) of the European Society for Blood and Marrow Transplantation (EBMT).

## Subjects and methods

### Study design and data collection

Eligibility criteria for this analysis included adult patients ≥18 years of age with sAML, mainly post-MDS/MPN, MPN, or MDS (Table 1) in CR1 who underwent a first haplo-HSCT with PTCy between 2010 and 2021. Additional information regarding study design and data collection is provided in Supplementary Appendix 1. The list of institutions contributing data to this study is provided in Supplementary Appendix 2.

**Table 1:** Patient and transplant characteristics.

Variable	Overall *N* = 554	BM *N* = 136	PBSC *N* = 418	*p*-value
**Year of transplantation, median (IQR)**	2018 (2016, 2020)	2017 (2015, 2019)	2019 (2017, 2020)	<0.001
**Median FU (y)**	3 (2.8–3.5)	4.1 (3.4–5.3)	2.8 (2.5–3)	
**Age of the patient at HSCT (y)**	61.2 (52.1, 67.1)	59.2 (50.4, 65.1)	61.7 (53.3, 67.4)	0.008
**Age of the donor at HSCT (y)**	37.2 (29.0, 45.8)	37.6 (29.9, 46.3)	37.1 (28.9, 45.7)	0.76
**Cytogenetic AML classification**				0.92
Favorable	5 (1.1%)	1 (1.0%)	4 (1.2%)	
Intermediate	280 (64.2%)	62 (63.3%)	218 (64.5%)	
Adverse	151 (34.6%)	35 (35.7%)	116 (34.3%)	
Missing	118	38	80	
**Previous diagnosis**				
MDS	150 (27%)	36 (26%)	114 (27%)	
MPN	33 (6.0%)	8 (5.9%)	25 (6.0%)	
MDS/MPN	259 (47%)	56 (41%)	203 (49%)	
Hematological malignancy	76 (14.3%)	26 (18.9%)	50 (11.8%)	
Solid tumor	33 (6.2%)	9 (6.6%)	25 (6%)	
Nonmalignant disease	1 (0.2%)	0 (0%)	1 (0.2%)	
Unknown	2 (0.4%)	1 (0.7%)	1 (0.2%)	
**Karnofsky score**				0.35
>= 90	380 (70.9%)	100 (74.1%)	280 (69.8%)	
< 90	156 (29.1%)	35 (25.9%)	121 (30.2%)	
Missing	18	1	17	
**HCT-CI**				0.074
0	214 (46.9%)	58 (56.9%)	156 (44.1%)	
1-2	92 (20.2%)	17 (16.7%)	75 (21.2%)	
>=3	150 (32.9%)	27 (26.5%)	123 (34.7%)	
Missing	98	34	64	
**Sex of the patient**				0.48
Female	226 (40.8%)	59 (43.4%)	167 (40.0%)	
Male	328 (59.2%)	77 (56.6%)	251 (60.0%)	
**Sex of the donor**				0.65
Female	216 (39.1%)	51 (37.5%)	165 (39.7%)	
Male	336 (60.9%)	85 (62.5%)	251 (60.3%)	
Missing	2	0	2	
**Female donor to male patient**				0.46
No	439 (79.4%)	111 (81.6%)	328 (78.7%)	
Yes	114 (20.6%)	25 (18.4%)	89 (21.3%)	
Missing	1	0	1	
**Patient CMV serostatus**				0.53
Negative	121 (22.1%)	27 (20.1%)	94 (22.8%)	
Positive	426 (77.9%)	107 (79.9%)	319 (77.2%)	
Missing	7	2	5	
**Donor CMV serostatus**				0.17
Negative	226 (41.5%)	48 (36.4%)	178 (43.2%)	
Positive	318 (58.5%)	84 (63.6%)	234 (56.8%)	
Missing	10	4	6	
**CMV antibodies donor to patient**				0.44
Neg to Neg	91 (16.9%)	17 (13.0%)	74 (18.1%)	
Neg to Pos	133 (24.6%)	31 (23.7%)	102 (24.9%)	
Pos to Neg	29 (5.4%)	9 (6.9%)	20 (4.9%)	
Pos to Pos	287 (53.1%)	74 (56.5%)	213 (52.1%)	
Missing	14	5	9	
**Type of conditioning**				<0.001
RIC	264 (48.0%)	45 (33.6%)	219 (52.6%)	
MAC	286 (52.0%)	89 (66.4%)	197 (47.4%)	
Missing	4	2	2	
**Months between diagnosis and HSCT**				0.019
Median	5.0	5.3	4.8	
(Q1, Q3)	(3.6, 6.7)	(4.0, 7.3)	(3.5, 6.6)	
Min, Max	1.1, 17.0	1.8, 16.2	1.1, 17.0	

*IQR* interquartile range, *y* years, *FU* follow up, *HSCT* hematopoietic stem cell transplantation, *BM* bone marrow, *PBSC* peripheral blood stem cells, *AML* acute myeloid leukemia, *CMV* cytomegalovirus, *neg* negative, *pos* positive, *HCT-CI* hematopoietic cell transplantation-specific comorbidity index, *MAC* myeloablative conditioning, *RIC* reduced intensity conditioning, *MDS* myelodysplastic syndrome, *MPN* myeloproliferative neoplasm.

Unless otherwise stated, results are expressed as frequency (%).

### Statistical analysis

The study endpoints were LFS, overall survival (OS), RI, NRM, the incidence of engraftment, aGVHD, cGVHD and GVHD-free, relapse-free survival (GRFS). All endpoints were measured from the time of transplantation. For univariable survival analysis, the Kaplan-Meier method was used to calculate OS, LFS, and GRFS. Cumulative incidence approach was used to estimate relapse incidence, NRM, aGVHD, cGvHD and poly and platelet recovery. Competing risks were death for relapse incidence and relapse for NRM, and relapse or death for aGVHD and cGVHD. Survival probabilities are given at 2 years as percentages and 95% confidence intervals (CIs). For multivariable analysis, a Cox proportional hazards model was performed including the following variables for adjustment: cell source, Karnofsky score, age of the patient (per 10 years) at HSCT, year of transplantation (per 5 years), female donor to male patient combination, cytogenetic AML classification, time (in months) between diagnosis and HSCT, type of conditioning, age of donor (per 10 years) and hematopoietic cell transplantation–specific comorbidity index (HCT-CI). To consider the heterogeneity in the effect of a characteristic or a treatment across centers, we introduced a random effect into the Cox multivariate models [28]. Hazard ratios (HR) were calculated together with corresponding 95% confidence intervals (95% CI). Significance were calculated using Wald test. The significance level was fixed at 0.05, and *p*-values were two-sided. *P*-values for secondary endpoints should be cautiously interpreted due to multiple comparisons. Additional statistical information is provided in Supplementary Appendix 3.

## Results

### Patient, transplant, and disease characteristics

In total, 554 patients met the inclusion criteria, 136 received BM and 418 PBSC as the stem cell source. Median follow-up was 3 years (95% CI: 2.8–3.5): 4.1 years (95% CI: 3.4–5.3) in the BM and 2.8 years (95% CI: 2.5–3) in the PBSC group (Table 1). The median year of transplant was 2018 (interquartile range [IQR]: 2016; 2020): 2017 (IQR: 2015; 2019) in the BM group and 2019 (IQR: 2017; 2020) in the PBSC (p < 0.001). Patients in the BM group were younger, median age 59.2 years (IQR: 50.4; 65.1) *vs*. 61.7 years (IQR: 53.3; 67.4) in the PBSC group (*p* = 0.008), with 56.6% and 60% being male, respectively (*p* = 0.48). The most common antecedent hematological disease was MDS/MPN being 41% *vs*. 49% in the BM and PBSC groups, followed by MDS, 26% *vs*. 27%, and MPN, 5.9% *vs*. 6%, respectively (Table 1). The cytogenetic risk groups, as classified by the European LeukemiaNet 2022 (ELN2022) were adverse in 35.7% *vs*. 34.3%, intermediate in 63.3% and 64.5%, and favorable in 1% and 1.2%, in the BM and PBSC groups, respectively (*p* = 0.92) (data were missing for 118 patients). Karnofsky Performance Status (KPS), HCT-CI, patient and donor CMV serostatus, donor age and gender, as well as female donor to male patient combination, did not differ between the groups. Time from diagnosis to haplo-HSCT was significantly longer in the BM *vs*. PBSC group, being 5.3 (IQR: 4.0; 7.3) and 4.8 (IQR: 3.5; 6.6) months, respectively (*p* = 0.019). Patients transplanted with PBSC received myeloablative conditioning (MAC) less frequently than patients receiving BM grafts (47.4% compared to 66.4%; *p* < 0.001) with thiotepa combined with busulfan and fludarabine being the most frequently used conditioning therapy in both groups (37.9% *vs*. 69.1% in the PBSC and BM groups, respectively) (Supplementary Table S1). The most frequent immunosuppression combined with PTCy as GVHD prophylaxis was mycophenolate mofetil with cyclosporine A (56% *vs*. 60%) or with tacrolimus (30.1% *vs*. 27.2%), respectively (Supplementary Table S2).

### Transplantation outcomes

**Fig. 1:**
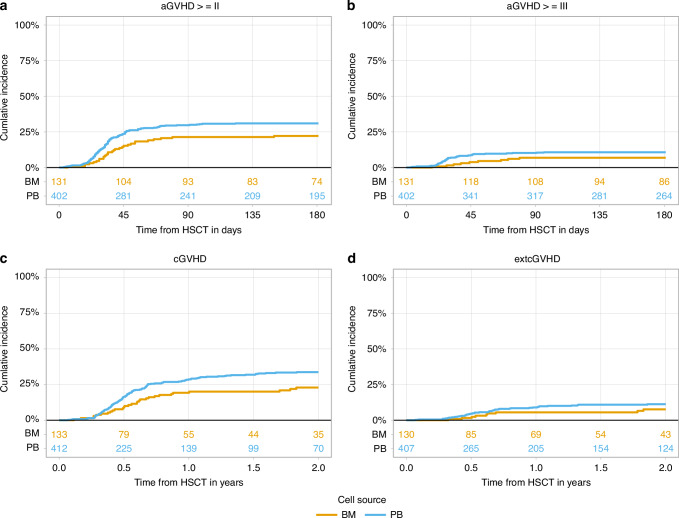
Acute and chronic graft-*versus-*host disease in sAML patients undergoing haplo-HSCT with PTCy from peripheral blood stem cells (PB) compared to bone marrow (BM) grafts. **a** aGVHD grade II-IV; **b** aGVHD grade III-IV; **c** cGVHD; **d** ext cGVHD. aGVHD acute graft-*versus*-host disease, cGVHD chronic graft-*versus*-host disease, ext extensive, BM bone marrow, PB peripheral blood, sAML secondary acute myeloid leukemia, haplo-HSCT haploidentical hematopoietic stem cell transplantation, PTCy post-transplant cyclophosphamide, aHR multivariable adjusted hazard ratio.

**Fig. 2:**
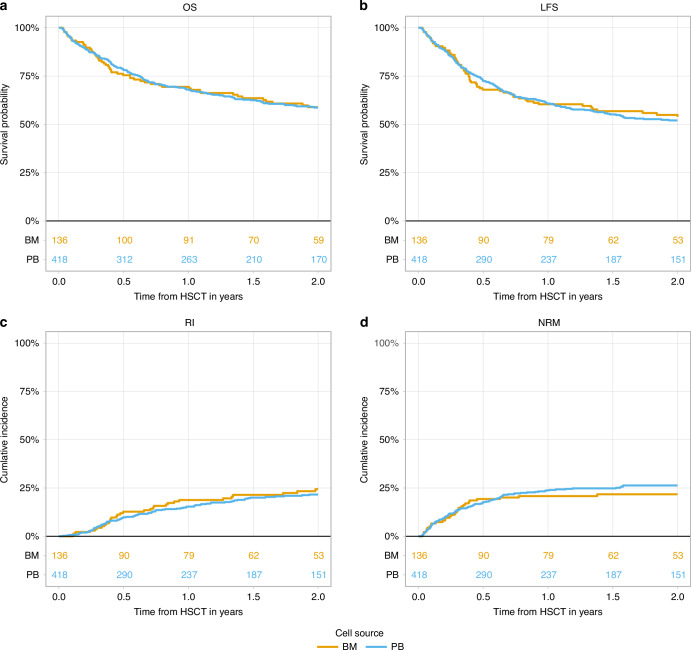
Transplantation outcome in sAML patients undergoing haplo-HSCT with PTCy from peripheral blood stem cells (PB) compared to bone marrow (BM) grafts. **a** OS; **b** LFS; **c** RI; **d** NRM. OS overall survival, LFS leukemia-free survival, RI relapse incidence, NRM non-relapse mortality, sAML secondary acute myeloid leukemia, BM bone marrow, PB peripheral blood, haplo-HSCT haploidentical hematopoietic stem cell transplantation, PTCy post-transplant cyclophosphamide, aHR multivariable adjusted hazard ratio.

The estimate (95% CI) of day 30 cumulative incidence of myeloid engraftment (ANC > 0.5 ×10^**9**^/L) was 88.1% (84.6–90.9%) *vs*. 83.5% (75.9–88.8%) of the patients in the PBSC and BM groups, respectively (Table 2). The median time from the transplant to ANC ≥ 1 ×10^9^/L was 20 (IQR: 17; 24) and 21 days (IQR: 18; 24), respectively. The estimate (95% CI) of day 60 cumulative incidence of platelet recovery (PLT ≥ 20 ×10^9^/L) was 79.8% (75.5–83.5%) and 75% (65.8–82.1%) in the PBSC and BM groups, respectively. The median time from the transplant to PLT ≥ 20 ×10^9^/L was 27 (IQR: 19; 37) and 30 days (IQR: 22; 42), respectively. The estimate (95% CI) of incidences of day 180 aGVHD, 2-year cGVHD and main transplant outcomes in both groups were shown in Table 2 and Figs. 1 and 2.

**Table 2:** Haploidentical hematopoietic stem cell transplantation outcomes.

Estimate (95% CI)	OS (2 y)	LFS (2 y)	GRFS (2 y)	RI (2 y)
**Overall (*N*** = **554)**	58.7 (54.3–62.9)	52.5 (48.1–56.7)	41.2 (36.9–45.5)	22.3 (18.8–26)
**BM (*N*** = **136)**	58.9 (49.7–66.9)	53.9 (44.8–62.1)	45.1 (36.1–53.7)	24.4 (17.2–32.2)
**PB (*N*** = **418)**	58.7 (53.6–63.4)	52 (46.9–56.9)	39.9 (35–44.8)	21.6 (17.7–25.9)

Estimate (95% CI)	NRM (2 y)	aGVHD >=II (180 d)	aGVHD >=III (180 d)	cGVHD (2 y)
**Overall (*N*** = **554)**	25.2 (21.6–28.9)	28.8 (25–32.7)	9.8 (7.4–12.5)	31.1 (27.1–35.1)
**BM (*N*** = **136)**	21.7 (15.1–29.1)	22.2 (15.5–29.6)	6.9 (3.4–12.1)	22.9 (15.9–30.6)
**PBSC (*N*** = **418)**	26.3 (22.1–30.7)	31 (26.5–35.5)	10.7 (7.9–14)	33.7 (29.1–38.5)

Estimate (95% CI)	Ext cGVHD (2 y)	Poly recovery (30 d)	Poly recovery (42 d)	Poly recovery (60 d)
**Overall (*N*** = **554)**	10.3 (7.9–13.2)	87 (83.9–89.5)	93 (90.5–94.9)	93.8 (91.4–95.5)
**BM (*N*** = **136)**	7.6 (3.7–13.4)	83.5 (75.9–88.8)	90.2 (83.7–94.2)	90.2 (83.7–94.2)
**PBSC (*N*** = **418)**	11.2 (8.3–14.6)	88.1 (84.6–90.9)	93.9 (91.1–95.9)	94.9 (92.2–96.7)

*y* year, *d* day, *BM* bone marrow, *PBSC* peripheral blood stem cells, *NRM* non-relapse mortality, *RI* relapse incidence, *LFS* leukemia-free survival, *OS* overall survival, *GVHD* graft-*versus*-host disease, *aGVHD * acute GVHD, *cGVHD* chronic GVHD, *Ext cGVHD* extensive chronic GVHD, *GRFS GVHD-free* relapse-free survival, Poly-polymorphonuclear.

Unless otherwise stated, results are expressed as frequency (%).

### Multivariate analysis

Table 3 shows the MVA results. The risk of aGVHD grades II-IV and III-IV as well as all grades and extensive cGVHD did not differ between patients receiving PBSC or BM as a graft source. There were also no differences between studied groups in other transplant outcomes including RI, NRM, LFS, OS and GFRS. A separate analysis conducted for patients receiving RIC and MAC regimens also revealed no significant differences in transplant outcomes between recipients of PBSC and BM grafts (Supplementary Tables S3, S4 and S5, and Figs. S1 and S2). Important factor that was significant in the MVA was lower KPS (score <90) that was a poor prognostic factor for NRM, OS, GRFS and LFS, while high HCT-CI was a risk factor for NRM, LFS and OS. Older age (per 10 years) was poor prognostic factor for LFS, NRM, OS and GRFS. Adverse-risk cytogenetics was also a poor prognostic factor for RI, OS and GRFS. It was also a predictive factor for all grades cGVHD: HR 1.93 (95% CI: 1.27–2.93, *p* = 0.002). In addition, there was an increased risk of all grades cGVHD in older patients (per 10 years), HR 1.25 (95% CI: 1.03–1.51, *p* = 0.021) and with a female donor to male patient combination, HR 2.09 (95% CI: 1.34–3.26, *p* = 0.001).

**Table 3:** Multivariate analysis.

	RELAPSE		NRM	
	HR (95% CI)	*p* value	HR (95% CI)	*p* value
**Cell source (reference BM)**	1.13 (0.68-1.86)	0.643	1.36 (0.78-2.38)	0.277
**Karnofsky score (reference >=90)**	0.86 (0.55-1.35)	0.508	0.59 (0.37-0.93)	0.024
**Age of the Patient at HSCT (per 10 years)**	1.05 (0.88-1.27)	0.57	1.34 (1.08-1.65)	0.007
**Year of transplantation per 5 years**	0.77 (0.49-1.21)	0.259	0.9 (0.55-1.48)	0.672
**Female donor to male patient (reference No)**	1.06 (0.65-1.73)	0.816	0.94 (0.58-1.53)	0.795
**Cytogenetic AML classification**	1.77 (1.18-2.66)	0.006	1.09 (0.68-1.75)	0.709
**Months between diagnosis and HSCT**	1 (0.92-1.07)	0.91	1.05 (0.98-1.12)	0.188
**Myeloablative regimen (reference No)**	0.85 (0.55-1.32)	0.468	1.28 (0.78-2.11)	0.334
**Age of the Donor at HSCT (per 10 years)**	0.91 (0.76-1.08)	0.263	1.06 (0.9-1.24)	0.503
**HCT-CI: 0 VS** > = **3**	1.4 (0.89-2.2)	0.149	1.65 (1.03-2.67)	0.039
**HCT-CI: 0 VS. 1-2**	1.14 (0.67-1.95)	0.634	1.32 (0.78-2.25)	0.301

	LFS		OS	
	HR (95% CI)	*p* value	HR (95% CI)	*p* value
**Cell source (reference BM)**	1.21 (0.85-1.74)	0.295	1.11 (0.75-1.63)	0.602
**Karnofsky score (reference >=90)**	0.67 (0.49-0.91)	0.01	0.65 (0.47-0.9)	0.01
**Age of the Patient at HSCT (per 10 years)**	1.14 (1-1.31)	0.049	1.27 (1.09-1.48)	0.002
**Year of transplantation per 5 years**	0.83 (0.59-1.15)	0.266	0.78 (0.54-1.11)	0.168
**Female donor to male patient (reference No)**	1.02 (0.73-1.44)	0.895	1.09 (0.76-1.56)	0.651
**Cytogenetic AML classification**	1.33 (0.98-1.79)	0.064	1.48 (1.07-2.03)	0.016
**Months between diagnosis and HSCT**	1.02 (0.97-1.07)	0.403	1.04 (0.98-1.09)	0.186
**Myeloablative regimen (reference No)**	1.12 (0.81-1.53)	0.493	1.15 (0.82-1.63)	0.418
**Age of the Donor at HSCT (per 10 years)**	1.01 (0.9-1.14)	0.825	1.02 (0.9-1.16)	0.716
**HCT-CI: 0 VS** > = **3**	1.43 (1.04-1.98)	0.029	1.53 (1.08-2.17)	0.018
**HCT-CI: 0 VS. 1-2**	1.24 (0.86-1.79)	0.258	1.2 (0.8-1.79)	0.381

	Acute GVHD II-IV		Acute GVHD III-IV	
	HR (95% CI)	*p* value	HR (95% CI)	*p* value
**Cell source (reference BM)**	1.45 (0.87-2.42)	0.155	1.56 (0.64-3.81)	0.332
**Karnofsky score (reference >=90)**	0.8 (0.53-1.22)	0.306	0.62 (0.3-1.25)	0.18
**Age of the Patient at HSCT (per 10 years)**	1.13 (0.94-1.36)	0.182	1.24 (0.89-1.74)	0.2
**Year of transplantation per 5 years**	1.32 (0.83-2.09)	0.245	1.32 (0.59-2.97)	0.498
**Female donor to male patient (reference No)**	0.73 (0.46-1.16)	0.19	0.68 (0.29-1.58)	0.369
**Cytogenetic AML classification**	1 (0.67-1.49)	0.994	1.32 (0.67-2.61)	0.429
**Months between diagnosis and HSCT**	0.98 (0.91-1.05)	0.502	0.98 (0.87-1.1)	0.733
**Myeloablative regimen (reference No)**	0.64 (0.42-0.97)	0.036	0.96 (0.46-2.02)	0.918
**Age of the Donor at HSCT (per 10 years)**	1.06 (0.91-1.24)	0.449	1.16 (0.9-1.49)	0.255
**HCT-CI: 0 VS** > = **3**	1.08 (0.69-1.67)	0.738	0.9 (0.41-1.98)	0.801
**HCT-CI: 0 VS. 1-2**	1.17 (0.73-1.88)	0.515	1.02 (0.45-2.3)	0.964

	chronic GVHD		extensive chronic GVHD	
	HR (95% CI)	*p* value	HR (95% CI)	*p* value
**Cell source (reference BM)**	1.7 (0.97-2.99)	0.065	1.15 (0.45-2.93)	0.773
**Karnofsky score (reference >=90)**	1.48 (0.9-2.42)	0.121	1.16 (0.54-2.47)	0.708
**Age of the Patient at HSCT (per 10 years)**	1.25 (1.03-1.51)	0.021	1.11 (0.8-1.52)	0.537
**Year of transplantation per 5 years**	0.82 (0.51-1.31)	0.397	1.28 (0.6-2.74)	0.52
**Female donor to male patient (reference No)**	2.09 (1.34-3.26)	0.001	1.82 (0.9-3.66)	0.094
**Cytogenetic AML classification**	1.93 (1.27-2.93)	0.002	1.8 (0.93-3.49)	0.079
**Months between diagnosis and HSCT**	1.01 (0.94-1.09)	0.732	1.05 (0.95-1.17)	0.358
**Myeloablative regimen (reference No)**	1.07 (0.67-1.71)	0.768	0.59 (0.27-1.29)	0.187
**Age of the Donor at HSCT (per 10 years)**	1.1 (0.93-1.3)	0.259	1.22 (0.93-1.6)	0.149
**HCT-CI: 0 VS** > = **3**	1.28 (0.8-2.05)	0.307	1.17 (0.55-2.49)	0.676
**HCT-CI: 0 VS. 1-2**	1.32 (0.8-2.2)	0.281	1.3 (0.56-3.05)	0.539

	GRFS	
	HR (95% CI)	*p* value
**Cell source (reference BM)**	1.22 (0.89-1.68)	0.224
**Karnofsky score (reference >=90)**	0.7 (0.54-0.92)	0.011
**Age of the Patient at HSCT (per 10 years)**	1.18 (1.04-1.33)	0.01
**Year of transplantation per 5 years**	0.93 (0.69-1.25)	0.62
**Female donor to male patient (reference No)**	1.1 (0.82-1.49)	0.521
**Cytogenetic AML classification**	1.36 (1.04-1.77)	0.023
**Months between diagnosis and HSCT**	1.01 (0.97-1.06)	0.575
**Myeloablative regimen (reference No)**	0.96 (0.73-1.27)	0.768
**Age of the Donor at HSCT (per 10 years)**	1.04 (0.94-1.15)	0.452
**HCT-CI: 0 VS** > = **3**	1.2 (0.9-1.6)	0.224
**HCT-CI: 0 VS. 1-2**	1.05 (0.75-1.46)	0.779

*NRM* non-relapse mortality, *LFS* leukemia-free survival, *OS* overall survival, *GVHD* graft-*versus*-host disease, *GRFS GVHD-free* relapse-free survival, *HR* hazard ratio, *CI* confidence interval, *BM* bone marrow, *HSCT* hematopoietic stem cell transplantation, *AML* acute myeloid leukemia.

### Cause of death

A total of 245 patients died during the study period, 183 (44%) in the PBSC group and 62 (48%) in the BM group. The main causes of death was the sAML or AML (38.3% *vs*. 50%) and infection (30.1% *vs*. 25.8%, respectively in the PBSC and BM group). All causes of death are shown in Table 4.

**Table 4:** Cause of death.

Variable	BM *N* = 62	PBSC *N* = 183
Secondary AML	31 (50.0%)	70 (38.3%)
Infection related	16 (25.8%)	55 (30.1%)
GVHD	5 (8.1%)	15 (8.2%)
Organ toxicity	5 (8.1%)	8 (4.4%)
GvHD + Infection related	2 (3.2%)	15 (8.2%)
Other transplant-related	1 (1.6%)	5 (2.7%)
Secondary malignancy	1 (1.6%)	1 (0.5%)
Other	1 (1.6%)	14 (7.7%)

*BM* bone marrow, *PBSC* peripheral blood stem cells, *GVHD* graft-*versus*-host disease, *AML* acute myeloid leukemia.

Results are expressed as frequency (%).

## Discussion

In this study, we have analysed transplant outcomes after haplo-HSCT with PTCy, comparing patients transplanted with PBSC *versus* those receiving BM grafts, focusing on sAML. In this setting we have shown no differences in main transplant outcomes between studied groups. Peripheral blood stem cells have become the main graft source in sibling and unrelated transplantation due to the well-known advantages of PBSC over BM as a graft source, such as convenient harvest, faster engraftment, lower incidence of graft failure and improved immune reconstitution, which, of course, may be offset by increased risk of chronic GVHD [29, 30]. On the other hand, cGVHD correlates with the GVL effect, which, although still controversial, may be stronger after transplantation with PBSC due to the higher numbers of cytotoxic T and natural killer cells and thus may advocate its use in high-risk acute leukemias like sAML [31, 32]. Although that also in haplo-HSCTs, which was pioneered with BM grafts and RIC [19], an increasing number of transplants are currently performed with PBSC [20, 21], the scenario may differ in the setting of haplo-HSCT with PTCy, given the strong tolerance induction involved and the dissociation of GVHD from GVL in haplo-HSCT with PTCy [33–35]. It is therefore that comparing PBSC *versus* BM as graft source for haplo-HSCT with PTCy in sAML is of interest and has clinical significance. Furthermore, as mentioned above, we have demonstrated that haplo-HSCT with PTCy may partially overcome the poor prognosis of sAML, rescuing 60% of patients while in remission but also 30% of those with relapsed/refractory disease [17, 18], suggesting that haplo-HSCT and thus optimizing the haplo-HSCT platform for sAML, choosing the optimal stem cell source was the obvious next question to address. Previous studies comparing PBSC to BM grafts for haplo-HSCT with PTCy yield some inconsistent results [22–26]. The current study results demonstrating no significant differences in NRM, RI, LFS, OS, and GRFS with PBSC and BM grafts as stem cell sources for haplo-HSCT with PTCy in sAML are similar to the results of the ALWP initial study comparing PBSC *versus* BM for haplo-HSCT in acute leukemia patients transplanted between 2010 and 2014 [23]. That initial study, published in 2018, included 451 patients with AML, mostly de novo AML, with only 17% having sAML, or acute lymphoblastic leukemia, demonstrated similar transplantation outcomes between the two graft sources, besides a lower incidence of aGVHD with BM grafts and better engraftment with PBSC grafts [23]. Similarly, the meta-analysis by Yu X and colleagues also demonstrated no difference in RI, LFS, and OS in haplo-HSCT with PTCy using PBSC compared to BM grafts, while aGVHD was higher and engraftment was better with PBSC grafts [24]. Mehta RS and colleagues from MD Anderson Cancer Center reported again no significant differences in NRM, RI, LFS, OS, and GRFS comparing PBSC *versus* BM grafts in patients with various hematologic malignancies undergoing haplo-HSCT with PTCy [25]. However, the incidence of total cGVHD was lower with BM grafts, as were the incidences of extensive and steroid-refractory cGVHD [25]. In another similar study comparing the two graft sources in patients with various hematological malignancies (34% AML) undergoing haplo-HSCT with PTCy, the only difference observed was a lower RI with PBSC, while all other transplantation outcomes were comparable with no significant differences [36]. An original study by the Center for International Blood and Marrow Transplant Research (CIBMTR) showed a lower RI with PBSC *versus* BM grafts in the setting of haplo-HSCT with PTCy. This study included 681 patients with various disease categories, and also showed lower acute and chronic GVHD with BM grafts and better GRFS with PBSC grafts [22]. The somewhat inconsistent results may be due to a difference in the various study cohorts, including differences in the underlying disease category (for example, the CIBMTR study cited above also included lymphoma patients), disease status at HSCT, transplant characteristics, and others. Notably and of major clinical importance in our current study we have showed that lower Karnofsky score is strongly associated with higher NRM and worse LFS, OS and GRFS. Similarly, high HCT-CI was a risk factor for NRM, LFS and OS and therefore those indices should be carefully assessed when qualifying patients to haplo-HSCT. This finding is in agreement with previous publications demonstrating the importance of patient performance status impacting transplantation outcomes [12, 17, and 18]. Adverse cytogenetics was found to be associated with higher risk of relapse and worse OS as previously described [12, 17, and 18]. Age and the intensity of the conditioning are two additional important parameters that are different in the various studies and may have affected the results. In our study, higher age (per 10 years) was a significant poor prognostic factor for NRM, LFS, OS, GRFS and the incidence of all grades of cGVHD, while RIC was associated with higher incidence of aGVHD grade II-IV. Of note, both patient age and conditioning intensity drastically impacted the results of some of the recently published PBSC *versus* BM comparisons in the PTCy-based haplo-HSCT setting [21, 26, 36]. The Société Francophone de Greffe de Moelle et de Thérapie Cellulaire (SFGM-TC) recently compared PBSC to BM grafts in 1344 patients with various hematological malignancies undergoing haplo-HSCT with PTCy, performing a subgroup analysis according to the intensity of the conditioning [21]. They demonstrated a higher RI in patients receiving RIC and BM *versus* PBSC grafts, while in patients receiving MAC, RI did not differ between those receiving BM compared to PBSC grafts [21]. In our study, when data were analyzed separately comparing PBSC versus BM grafts in patients receiving either MAC or RIC we could not demonstrate significant differences in transplantation outcomes. Baron et al. on behalf of the ALWP, compared PBSC *versus* BM grafts in 668 patients with relapsed/refractory AML undergoing haplo-HSCT with PTCy. Results differed in patients older or younger than 55 years of age. In patients ≤55 years, transplantation outcomes were similar, while in patients >55 years, NRM was higher and LFS and OS were lower with PBSC grafts [37]. Notably, different results were observed by Devillier et al. who recently compared PBSC to BM grafts in a large group (*n* = 804) of AML patients >60 years who were transplanted in CR from haploidentical donors with PTCy anti-GVHD prophylaxis and with RIC, demonstrating lower risk of relapse and better LFS in patients transplanted with PBSC compared to BM grafts [26]. Finally, in a very recent analysis by the CIBMTR, the authors investigated the outcomes following BM *versus* PBSC haplo-HSCT using PTCy-based GVHD prophylaxis in 550 patients with various hematological malignancies (54% AML). In that study the recipients of PBSC grafts had a higher incidence of cGVHD and slightly lower GRFS compared to those with BM grafts, while other transplantation outcomes, including OS, disease-free survival, RI, NRM, and aGVHD, were comparable [38]. As for sAML there are almost no previous studies comparing PBSC to BM grafts focusing on this specific AML category. We previously compared the two graft sources in a small group of patients with sAML (*n* = 154) that underwent haplo-HSCT between 2006 and 2016, allowing for both anti-thymocyte globulin (ATG) and PTCy [39]. The use of BM as a graft source was associated with higher RI and lower NRM when compared to PBSC, while severe aGVHD was observed more frequently in the latter. However, these differences did not meet statistical significance in the MVA [39]. The use of ATG could account for the somewhat different results as compared to our current study. In that previous study, we demonstrated significantly better results with PTCy anti-GVHD prophylaxis in comparison to ATG-based GVHD prophylaxis; the use of ATG was associated with inferior NRM, LFS, OS, and GRFS [39]. The current study focuses on a rather homogenous sAML patient population undergoing haplo-HSCT with PTCy in CR1, thus an important step forward.

Our current study, being registry-based, has some limitations, including the lack of information on frontline therapies, number of infused CD34 + /CD3+ cells in grafts as well as missing data on measurable residual disease or comorbidities that might have influenced the outcomes. Moreover, the lack of molecular data precluded a comprehensive classification of risk categories according to the ELN2022. However, in this real-life setting comparing PBSC to BM as graft source for haplo-HSCT with PTCy in a large homogeneous population of sAML, we have demonstrated comparable main transplant outcomes in patients receiving both types of grafts. Although the final answer to the question of “what is the optimal and preferable graft for haplo-HSCT with PTCy in patients with sAML” necessitates well-designed randomized, prospective studies, the chance of such studies being performed in the near future in sAML is rather small, and until then, our study is not just valid but is unique.

## Supplementary information

Supplementary Appendices 1-3

Supplementary Figures S1-S2

Supplementary Tables S1-S5

## Data Availability

AN, ATF, MM and FC had full access to all study data (available upon data-specific request).

## References

[1] Lindsley RC, Mar BG, Mazzola E, Grauman PV, Shareef S, Allen SL, et al. Acute myeloid leukemia ontogeny is defined by distinct somatic mutations. Blood. 2015;125:1367–76. 25550361 10.1182/blood-2014-11-610543 PMC4342352

[2] Granfeldt Ostgard LS, Medeiros BC, Sengelov H, Norgaard M, Andersen MK, Dufva IH, et al. Epidemiology and clinical significance of secondary and therapy-related acute myeloid leukemia: A National Population-Based Cohort Study. J Clin Oncol. 2015;33:3641–9. 26304885 10.1200/JCO.2014.60.0890

[3] Hulegårdh E, Nilsson C, Lazarevic V, Garelius H, Antunovic P, Rangert Derolf Å, et al. Characterization and prognostic features of secondary acute myeloid leukemia in a population-based setting: A report from the Swedish Acute Leukemia Registry. Am J Hematol. 2015;90:208–14. 25421221 10.1002/ajh.23908

[4] Winer ES. Secondary Acute Myeloid Leukemia: A Primary Challenge of Diagnosis and Treatment. Hematol Oncol Clin North Am. 2020;34:449–63. 32089222 10.1016/j.hoc.2019.11.003

[5] Dunbar AJ, Rampal RK, Levine R. Leukemia secondary to myeloproliferative neoplasms. Blood. 2020;136:61–70. 32430500 10.1182/blood.2019000943 PMC7332899

[6] Döhner H, Wei AH, Appelbaum FR, Craddock C, Dinardo CD, Dombret H, et al. Diagnosis and management of AML in adults: 2022 ELN recommendations from an international expert panel on behalf of ELN. Blood. 2022;140:1345–77. 35797463 10.1182/blood.2022016867

[7] Döhner H, DiNardo CD, Appelbaum FR, Craddock C, Dombret H, Ebert BL, et al. Genetic risk classification for adults with AML receiving less-intensive therapies: the 2024 ELN recommendations. Blood. 2024;144:2169–73. 39133932 10.1182/blood.2024025409

[8] Tazi Y, Arango-Ossa JE, Zhou Y, Bernard E, Thomas I, Gilkes A, et al. Unified classification and risk-stratification in Acute Myeloid Leukemia. Nat Commun. 2022;13:4622. 35941135 10.1038/s41467-022-32103-8 PMC9360033

[9] Uy GL, Newell LF, Lin TL, Goldberg SL, Wieduwilt MJ, Ryan RJ, et al. Transplant outcomes after CPX-351 vs 7 + 3 in older adults with newly diagnosed high-risk and/or secondary AML. Blood Adv. 2022;6:4989–93. 35443022 10.1182/bloodadvances.2021006468 PMC9631647

[10] Litzow MR, Tarima S, Perez WS, Bolwell BJ, Cairo MS, Camitta BM, et al. Allogeneic transplantation for therapy-related myelodysplastic syndrome and acute myeloid leukemia. Blood. 2010;115:1850–7. 20032503 10.1182/blood-2009-10-249128 PMC2832815

[11] Sengsayadeth S, Labopin M, Boumendil A, Finke J, Ganser A, Stelljes M, et al. Transplant Outcomes for Secondary Acute Myeloid Leukemia: Acute Leukemia Working Party of the European Society for Blood and Bone Marrow Transplantation Study. Biol Blood Marrow Transpl. 2018;24:1406–14. 10.1016/j.bbmt.2018.04.008 29678639

[12] Schmaelter A-K, Labopin M, Socié G, Itälä-Remes M, Blaise D, Yakoub-Agha I, et al. Inferior outcome of allogeneic stem cell transplantation for secondary acute myeloid leukemia in first complete remission as compared to de novo acute myeloid leukemia. Blood Cancer J. 2020;10:26. 32127519 10.1038/s41408-020-0296-3 PMC7054545

[13] Nagler A, Ngoya M, Jacques-Emmanuel G, Labopin M, Kröger N, Socié G, et al. Trends in the outcome of transplantation in patients with secondary acute myeloid leukemia: an analysis from the Acute Leukemia Working Party (ALWP) of the EBMT. Bone Marrow Transpl. 2022;57:1788–96. 10.1038/s41409-022-01825-0 36114249

[14] Luznik L, O’Donnell PV, Fuchs EJ. Post-transplantation cyclophosphamide for tolerance induction in HLA-haploidentical bone marrow transplantation. Semin Oncol. 2012;39:683–93. 23206845 10.1053/j.seminoncol.2012.09.005 PMC3808078

[15] McCurdy SR, Kanakry JA, Showel MM, Tsai HL, Bolanos-Meade J, Rosner GL, et al. Risk-stratified outcomes of nonmyeloablative HLA-haploidentical BMT with high-dose post-transplantation cyclophosphamide. Blood. 2015;125:3024–31. 25814532 10.1182/blood-2015-01-623991 PMC4424420

[16] Guo H, Chang YJ, Hong Y, Xu LP, Wang Y, Zhang XH, et al. Dynamic immune profiling identifies the stronger graft-versus-leukemia (GVL) effects with haploidentical allografts compared to HLA-matched stem cell transplantation. Cell Mol Immunol. 2021;18:1172–85. 33408344 10.1038/s41423-020-00597-1 PMC8093297

[17] Nagler A, Labopin M, Blaise D, Raiola AM, Corral LL, Bramanti S, et al. Non-T-depleted haploidentical transplantation with post-transplant cyclophosphamide in patients with secondary versus de novo AML in first complete remission: a study from the ALWP/EBMT. J Hematol Oncol. 2023;16:58. 37248463 10.1186/s13045-023-01450-4 PMC10226209

[18] Nagler A, Labopin M, Tischer J, Raiola AM, Kunadt D, Vydra J, et al. Haploidentical transplantation in primary refractory/relapsed secondary vs de novo AML: from the ALWP/EBMT. Blood Adv. 2024;8:4223–33. 38598754 10.1182/bloodadvances.2024012798 PMC11372397

[19] Luznik L, O’Donnell PV, Symons HJ, Chen AR, Leffell MS, Zahurak M, et al. HLA-haploidentical bone marrow transplantation for hematologic malignancies using nonmyeloablative conditioning and high-dose, post-transplantation cyclophosphamide. Biol Blood Marrow Transpl. 2008;14:641–50. 10.1016/j.bbmt.2008.03.005 PMC2633246 18489989

[20] Passweg JR, Baldomero H, Atlija M, Kleovoulou I, Witaszek A, Alexander T, et al. The 2023 EBMT report on hematopoietic cell transplantation and cellular therapies. Increased use of allogeneic HCT for myeloid malignancies and of CAR-T at the expense of autologous HCT. Bone Marrow Transpl. 2025;60:519–28. 10.1038/s41409-025-02524-2 PMC11971038 39939433

[21] Lacan C, Lambert J, Forcade E, Robin M, Chevallier P, Loron S, et al. Bone marrow graft versus peripheral blood graft in haploidentical hematopoietic stem cells transplantation: a retrospective analysis in 1344 patients of the SFGM-TC registry. J Hematol Oncol. 2024;17:2. 38185663 10.1186/s13045-023-01515-4 PMC10773006

[22] Bashey A, Zhang MJ, McCurdy SR, St Martin A, Argall T, Anasetti C, et al. Mobilized peripheral blood stem cells versus unstimulated bone marrow as a graft source for T-Cell-replete haploidentical donor transplantation using post-transplant cyclophosphamide. J Clin Oncol. 2017;35:3002–9. 28644773 10.1200/JCO.2017.72.8428 PMC5590802

[23] Ruggeri A, Labopin M, Bacigalupo A, Gülbas Z, Koc Y, Blaise D, et al. Bone marrow versus mobilized peripheral blood stem cells in haploidentical transplants using post-transplantation cyclophosphamide. Cancer. 2018;124:1428–37. 29360162 10.1002/cncr.31228

[24] Yu X, Liu L, Xie Z, Dong C, Zhao L, Zhang J, et al. Bone marrow versus peripheral blood as a graft source for haploidentical donor transplantation in adults using post-transplant cyclophosphamide: A systematic review and meta-analysis. Crit Rev Oncol Hematol. 2019;133:120–8. 30661648 10.1016/j.critrevonc.2018.05.017

[25] Mehta RS, Saliba RM, Alsfeld LC, Jorgensen JL, Wang SA, Anderlini P, et al. Bone marrow versus peripheral blood grafts for haploidentical hematopoietic cell transplantation with post-transplantation cyclophosphamide. Transpl Cell Ther. 2021;27:1003.e1–1003.e13. 10.1016/j.jtct.2021.09.003 PMC8504778 34537419

[26] Devillier R, Galimard JE, Blaise D, Raiola AM, Bramanti S, Grillo G, et al. Peripheral blood stem cell versus bone marrow graft for patients ≥60 years undergoing reduced intensity conditioning haploidentical transplantation for acute myeloid leukemia in complete remission: An analysis of the Acute Leukemia Working Party of the European Society for Blood and Marrow Transplantation. Am J Hematol. 2024;99:1250–6. 38778766 10.1002/ajh.27343

[27] Martínez-Cuadrón D, Megías-Vericat JE, Serrano J, Martínez-Sánchez P, Rodríguez-Arbolí E, Gil C, et al. Treatment patterns and outcomes of 2310 patients with secondary acute myeloid leukemia: a PETHEMA registry study. Blood Adv. 2022;6:1278–95. 34794172 10.1182/bloodadvances.2021005335 PMC8864639

[28] Kanate AS, Nagler A, Savani B. Summary of Scientific and Statistical Methods, Study Endpoints and Definitions for Observational and Registry-Based Studies in Hematopoietic Cell Transplantation. Clin Hematol Int. 2019;2:2–4. 34595436 10.2991/chi.d.191207.001 PMC8432337

[29] Anasetti C, Logan BR, Lee SL, Waller EK, Weisdorf DJ, Wingard JR, et al. Peripheral-blood stem cells versus bone marrow from unrelated donors. N Engl J Med. 2012;367:1487–96. 23075175 10.1056/NEJMoa1203517 PMC3816375

[30] Savani BN, Labopin M, Blaise D, Niederwieser D, Ciceri F, Ganser A, et al. Peripheral blood stem cell graft compared to bone marrow after reduced intensity conditioning regimens for acute leukemia: a report from the ALWP of the EBMT. Haematologica. 2016;101:256–62. 26565001 10.3324/haematol.2015.135699 PMC4938329

[31] Maurer K, Soiffer RJ. The delicate balance of graft versus leukemia and graft versus host disease after allogeneic hematopoietic stem cell transplantation. Expert Rev Hematol. 2023;16:943–62. 37906445 10.1080/17474086.2023.2273847 PMC11195539

[32] Weisdorf DJ. Which donor or graft source should you choose for the strongest GVL? Is there really any difference? Best. Pr Res Clin Haematol. 2013;26:293–6. 10.1016/j.beha.2013.10.010 PMC3900021 24309533

[33] Shimoni A, Peczynski C, Labopin M, Kulagin A, Meijer E, Cornelissen J, et al. Post-transplant cyclophosphamide separates graft-versus-host disease and graft-versus-leukemia effects after HLA-matched stem-cell transplantation for acute myeloid leukemia. Leukemia. 2025;39:222–8. 39482353 10.1038/s41375-024-02445-x PMC11717700

[34] Shimoni A, Labopin M, Angelucci E, Blaise D, Ciceri F, Koc Y, et al. The association of graft-versus-leukemia effect and graft-versus-host disease in haploidentical transplantation with post-transplant cyclophosphamide for AML. Bone Marrow Transpl. 2022;57:384–90. 10.1038/s41409-021-01493-6 35022535

[35] Baron F, Labopin M, Tischer J, Raiola AM, Vydra J, Blaise D, et al. GVHD occurrence does not reduce AML relapse following PTCy-based haploidentical transplantation: a study from the ALWP of the EBMT. J Hematol Oncol. 2023;16:10. 36782226 10.1186/s13045-023-01403-x PMC9923893

[36] Sharma N, Faisal MS, Zhao Q, Jiang J, Edler P, Benson DM, et al. Outcomes of bone marrow compared to peripheral blood for haploidentical transplantation. J Clin Med. 2021;10:2843. 34199028 10.3390/jcm10132843 PMC8268935

[37] Baron F, Labopin M, Tischer J, Ciceri F, Raiola AM, Blaise D, et al. Human leukocyte antigen-haploidentical transplantation for relapsed/refractory acute myeloid leukemia: Better leukemia-free survival with bone marrow than with peripheral blood stem cells in patients ≥55 years of age. Am J Hematol. 2022;97:1065–74. 35696192 10.1002/ajh.26627

[38] Mushtaq M, Kasaeian A, Chaudhary SG, Amin MK, Khavandgar N, Alemi H, et al. Outcomes after bone marrow versus peripheral blood haploidentical hematopoietic cell transplantation using posttransplant cyclophosphamide-based GVHD prophylaxis. Blood. 2024;144:4877.

[39] Li Z, Labopin M, Ciceri F, Blaise D, Tischer J, Ehninger G, et al. Haploidentical transplantation outcomes for secondary acute myeloid leukemia: Acute Leukemia Working Party (ALWP) of the European Society for Blood and Marrow Transplantation (EBMT) study. Am J Hematol. 2018;9:769–77. 10.1002/ajh.25087 29536560

