# Supplementary material for: Peripheral blood stem cells versus bone marrow graft for non-T-depleted haploidentical transplantation with post-transplant cyclophosphamide in patients with secondary acute myeloid leukemia in first complete remission: A study from the ALWP/EBMT
Source: Bone Marrow Transplant. 2026 Mar 25;61(5):559–68. doi: 10.1038/s41409-026-02823-2 (PMC13152805; doi:10.1038/s41409-026-02823-2)
Supplement: Supplementary file 1 — Supplementary Appendices 1-3 [file 41409_2026_2823_MOESM1_ESM.docx]

**Supplementary Appendix 1: Subjects and Methods**

*Study design and data collection*

Secondary AML was defined as AML in patients with a history of MDS, MPN, MDS/MPN, other hematological malignancy, solid tumor or aplasia.

The graft source was either PBSC or BM. Both RIC and myeloablative conditioning (MAC) were permitted. The exclusion criteria were HSCT from other donor types (MSD, MUD, or cord blood), previous history of HSCT, and *ex vivo* or *in vivo* T-cell depletion.

Data collected included recipient and donor characteristics [age, gender, cytomegalovirus (CMV) serostatus, and Karnofsky performance status (KPS)], hematopoietic cell transplantation-specific comorbidity index (HCT-CI), disease characteristics, year of transplant, type of conditioning regimen, stem cell source, and GVHD prophylaxis regimen. The conditioning regimen was defined as MAC when containing total body irradiation with a dose >6 Gray or a total dose of busulfan >8 mg/kg or >6.4 mg/kg when administered orally or intravenously, respectively, or total dose of treosulfan >36 gr/m^2^. All other regimens were defined as RIC [40]. Grading of acute (a) GVHD was performed using established criteria [41]. Chronic (c) GVHD was classified as limited or extensive according to published criteria [42]. For this study, all necessary data were collected according to the EBMT guidelines, using the EBMT minimum essential data forms.

**Supplementary Appendix 2: Contributing Centers**

Programme de Transplantation & Therapie Cellulaire, Marseille, France; Medicana International Hospital Istanbul, Istanbul, Turkey; Ospedale San Raffaele s.r.l., Rome, Italy; IRCCS Ospedale Policlinico San Martino, Genova, Italy; S.S.C.V.D Trapianto di Cellule Staminali, Torino, Italy; Imperial College Hammersmith London, London, United Kingdom; Istituto Clinico Humanitas, Milano, Italy; Turku University Hospital, Turku, Finland; Hospital U. Marqués de Valdecilla, Santander, Spain; Institute of Hematology and Blood Transfusion, Prague, Czech Republic; RM Gorbacheva Research Institute, Pavlov University, Petersburg, Russian Federation; Département d'Oncologie, Service d'Hématologie, Geneva, Switzerland; CHU Bordeaux, Hopital Haut-Leveque, Pessac, France; ASST Grande Ospedale Metropolitano Niguarda, Milano, Italy; Hospital Ramón y Cajal, Madrid, Spain; Azienda Ospedali Riuniti di Ancona, Ancona, Italy; Universitaetsklinikum Dresden, Dresden, Germany; Hospital Gregorio Marañón, Madrid, Spain; Hospital Clinic, Barcelona, Spain; European Institute of Oncology, Milano, Italy; Secretary and Italian National BMT Registry - GITMO, Bergamo, Italy; Hospital Clínico, Salamanca, Spain; Klinikum Augsburg, Augsburg, Germany; Universita Cattolica S. Cuore, Rome, Italy; ALBERTS CELLULAR THERAPY, Pretoria, South Africa; Universitaetsmedizin Mannheim, Mannheim, Germany; Saint-Louis Hospital, BMT Unit, Paris, France; CHU de Lille, Lille, France; University Hospital Frankfurt - Goethe University, Frankfurt Main, Germany; CHU Nice - Hôpital de l`Archet I, Nice, France; AORMN Hospital, Pesaro, Italy; University Medical Center Groningen (UMCG), Groningen, Netherlands; Arcispedale S. Maria Nuova, Reggio E, Italy; Demiroglu Bilim University Istanbul Florence Nightingale Hospital, Istanbul, Turkey; USD Trapianti di Midollo, Adulti, Brescia, Italy; Medizinische Universitaet Wien, Vienna, Austria; Oslo University Hospital, Rikshospitalet, Oslo, Norway; Hospital Santa Creu i Sant Pau, Barcelona, Spain; Universite Paris IV, Hopital la Pitié-Salpêtrière, Paris, France; CHU Grenoble Alpes - Université Grenoble Alpes, Grenoble, France; RVI Newcastle, Newcastle, United Kingdom; Hospital Clínico de Valencia, Valencia, Spain; Hannover Medical School, Hannover, Germany; Anadolu Medical Center Hospital, Kocaeli, Turkey; Grande Ospedale Metropolitano Bianchi Melacrino Morelli - Centro Unico Trapianti A. Neri, Reggio Calabria, Italy; University Hospital Eppendorf, Hamburg, Germany; Policlinico G.B. Rossi, Verona, Italy; AZ Delta, Roeselare, Belgium; University Hospital La Fe, Valencia, Spain; Centre Hospitalier Lyon Sud, Lyon, France; Azienda Ospedaliero Universitaria di Udine, Udine, Italy; University of Napoli, Napoli, Italy; Unita Operativa di Ematologia e Trapianto di cellule staminali, Lecce, Italy; San Matteo Pavia Transplant Programme, Pavia, Italy; Hospital Sirio-Libanes, Sao Paulo, Brazil; Institut Jules Bordet, Brussels, Belgium; University Hospital | Essen, Essen, Germany; Hopital La Miletrie, Poitiers, France; Ospedale S. Camillo-Forlanini, Rome, Italy; University Hospital Center Rebro, Zagreb, Croatia; University Hospitals Bristol and Weston NHSFT, Bristol, United Kingdom; Klinikum Grosshadern, Munich, Germany; University Hospital Maastricht, Maastricht, Netherlands; Elisabethinen-Hospital, Linz, Austria; Az. Ospedaliera S. Croce e Carle, Cuneo, Italy; ICO-Hospital Universitari Germans Trias i Pujol, Badalona, Spain; Philipps Universitaet Marburg, Marburg, Germany; ICANS - Institut de cancérologie Strasbourg Europe, Strasbourg, France; Kings College Hospital London, London, United Kingdom; A.O.R.N. `SAN.G MOSCATI`, Avellino, Italy; Azienda Ospedaliero Universitaria Pisana, Pisa, Italy; University Clinical Centre in Gda?sk, Gdansk, Poland; University Hospital Erlangen, Erlangen, Germany; Centro Trapianti Unico Di CSE Adulti e Pediatrico A. O Brotzu, Cagliari, Italy; Antwerp University Hospital (UZA), Antwerp E, Belgium; Gazi University Faculty of Medicine, Ankara, Turkey; Klinikum Frankfurt (Oder) GmbH, Frankfurt Oder, Germany; University Hospital | Basel, Basel, Switzerland; University Hospital, Zürich, Switzerland; University College London Hospital, London, United Kingdom; Institut de Cancerologie Lucien Neuwirth, Saint Etienne, France; CHU CAEN, Caen, France; Fondazione IRCCS – Ca’ Granda, Milano, Italy; ASCTR - Austrian Stem Cell Transplantation Registry, Innsbruck, Austria; Birmingham Centre for Cellular Therapy and Transplant (BCCTT), Birmingham, United Kingdom; Sahlgrenska University Hospital, Goeteborg, Sweden; Klinikum Karlsruhe gGmbH, Karlsruhe, Germany; Hospital San Maurizio, Bolzano, Italy; Martin-Luther-Universitaet Halle-Wittenberg, Halle, Germany; HUCH Comprehensive Cancer Center, Helsinki, Finland; IRCCS, Casa Sollievo della Sofferenza, San Giovanni, Italy; Ospedale San Gerardo, Monza, Italy; George Papanicolaou General Hospital, Thessaloniki, Greece; Cardarelli Hospital, Napoli, Italy; Evangelismos Hospital, Athens, Greece; Universitair Ziekenhuis Brussel, Brussels, Belgium; C.H.R.U de Brest, Brest, France; Gustave Roussy Cancer Campus, Villejuif, France; CHRU NANCY, Vandoeuvre les Nancy, France; Clinica Puerta de Hierro, Madrid, Spain; Umea University Hospital, Umeå, Sweden; Hospital Morales Meseguer, Murcia, Spain; Hospital Universitario Virgen del Rocío, Sevilla, Spain; University of Saarland, Homburg, Germany; University Regensburg, Regensburg, Germany; Ospedale San Carlo, Potenza, Italy; Perrino Hospital, Brindisi, Italy; Medical School University of Salerno, Salerno, Italy; Centre Henri Becquerel, Rouen, France; CHRU Limoges, Limoges, France; Robert_Bosch_Krankenhaus, Stuttgart, Germany; University Hospital Ostrava, Ostrava, Czech Republic; Hopital Necker Adults, Paris, France; Karolinska University Hospital, Stockholm, Sweden; Royal Marsden Hospital, London, United Kingdom; Hopital Jean Minjoz, Besancon, France; Hospital de la Princesa, Madrid, Spain; Nijmegen Medical Centre, Nijmegen, Netherlands; Bologna University, S.Orsola-Malpighi Hospital, Bologna, Italy; Ospedale Civile, Pescara, Italy; CHU Nantes, Nantes, France; CHU ESTAING, Clermont, France; Azienda Ospedaliera Universitaria Careggi, Firenze, Italy; Fundación Jiménez Díaz, Madrid, Spain; U.O.S.A Centro Trapianti e Terapia Cellulare, Siena, Italy; ZNA, Antwerp, Belgium; Hospital Álvaro Cunqueiro - Complejo Hospitalario Universitario de Vigo, Vigo, Spain; Fundeni Clinical Institute, Bucharest, Romania; King Fahad Specialist Hospital, Dammam, Saudi Arabia; King Abdul - Aziz Medical City, Riyadh, Saudi Arabia; University of Cologne, Cologne, Germany; Hospital de Gran Canaria Dr Negrin, Las Palmas, Spain; Azienda Ospedaliero Universitaria di Modena Policlinico, Modena, Italy; Dél-pesti Centrumkórház, Budapest, Hungary; Klinikum Rechts der Isar, Munich, Germany; Addenbrookes Hospital Cambridge, Cambridge, United Kingdom; Hospital Regional de Málaga, Malaga, Spain; Hospital Universitario de Navarra, Pamplona, Spain; King Hussein Cancer Centre Adult BMT Program, Amman, Jordan; Hospital Vall d`Hebron, Barcelona, Spain; CHU - Institut Universitaire du Cancer Toulouse, Toulouse, France; University of Debrecen Clinical Center, Debrecen, Hungary; CHRU, Angers, France; Centre Hospitalier Universitaire de Rennes, Rennes, France; Nottingham City Hospital, Nottingham, United Kingdom; Charles University Hospital, Pilsen, Czech Republic; University of Liege, Liege, Belgium; Ghent University Hospital, Gent, Belgium; Institut Catalá d`Oncologia,  Hospital Duran i Reynals, Barcelona, Spain; St. Bartholomew`s Hospital London, London, United Kingdom; University Medical Center Mainz, Mainz, Germany; S. Bortolo Hospital, Vicenza, Italy; H SS. Antonio e Biagio, Alessandria, Italy;

**Supplementary Appendix 3: Statistical analysis**

Overall survival (OS) was defined as the duration from the start of the study to death from any cause. Leukemia-free survival (LFS) was defined as the time to either relapse or death, in competition. GVHD-free-relapse-free survival (GRFS) was defined as the time to the first occurrence of any of the following events: grade III-IV acute graft-versus-host disease (aGVHD), extensive chronic graft-versus-host disease (cGVHD), relapse, or death from any cause [43]. The diagnosis and grading of acute and chronic GVHD were conducted according to the revised criteria of the Mount Sinai International Consortium and the National Institutes of Health (NIH), respectively. Engraftment was defined as an absolute neutrophil count (ANC) of 0.5×10^9^/L for three consecutive days after transplantation. NRM was defined as death from any cause without previous relapse or progression. Numerical data between studied groups were compared using the Mann–Whitney *U* test, while the chi-squared or Fisher’s exact test was used for categorical data. Statistical analyses and adjusted survival curves were performed using the R statistical software version 4.2.3 (R Foundation for Statistical Computing, Austria, Vienna; available online at http://www.R-project.org).

**References:**

40. Bacigalupo A, Ballen K, Rizzo D, Giralt S, Lazarus H, Ho V, et al. Defining the intensity of conditioning regimens: working definitions. Biol Blood Marrow Transplant. 2009; 15:1628-1633.

41. Przepiorka D, Weisdorf D, Martin P, Klingemann HG, Beatty P, Hows J, et al. Consensus Conference on Acute GVHD Grading. Bone Marrow Transplant.1995; 15:825-828.

42. Shulman HM, Sullivan KM, Weiden PL, McDonald GB, Striker GE, Sale GE, et al. Chronic graft-versus-host syndrome in man. A long-term clinicopathologic study of 20 Seattle patients. Am J Med. 1980; 69:204-217.

43. Ruggeri A, Labopin M, Ciceri F, Mohty M, Nagler A. Definition of GvHD-free, relapse-free survival for registry-based studies: an ALWP-EBMT analysis on patients with AML in remission. Bone marrow transplant. 2016; 51:610-611.
