# Supplementary material for: Peripheral blood stem cells versus bone marrow graft for non-T-depleted haploidentical transplantation with post-transplant cyclophosphamide in patients with secondary acute myeloid leukemia in first complete remission: A study from the ALWP/EBMT
Source: Bone Marrow Transplant. 2026 Mar 25;61(5):559–68. doi: 10.1038/s41409-026-02823-2 (PMC13152805; doi:10.1038/s41409-026-02823-2)

**Supplementary Figure S1. Transplantation outcome in sAML patients undergoing haplo-HSCT with PTCy from peripheral blood stem cells (PB) compared to bone marrow (BM) grafts: reduced intensity conditioning compared to myeloablative condition (RIC versus MAC)**

RIC-reduced intensity conditioning; MAC-myeloablative conditioning; OS – overall survival; LFS – leukemia-free survival; RI – relapse incidence; NRM – non-relapse mortality; BM – bone marrow; PB – peripheral blood; HSCT-hematopoietic stem cell transplantation


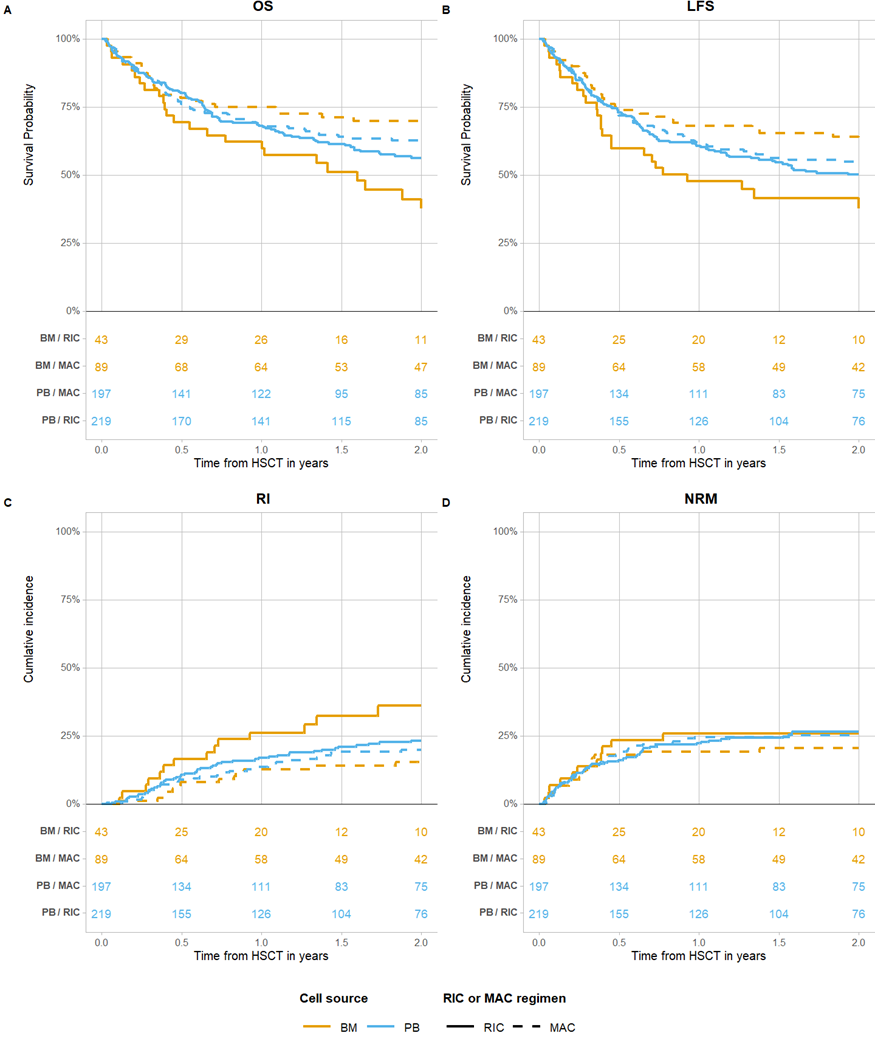


**Supplementary Figure S2. Acute and chronic graft-versus-host disease in sAML patients undergoing haplo-HSCT with PTCy from peripheral blood stem cells (PB) compared to bone marrow (BM) grafts: reduced intensity conditioning compared to myeloablative condition (RIC versus MAC)**

RIC-reduced intensity conditioning; MAC-myeloablative conditioning; aGVHD – acute graft-*versus*-host disease; cGVHD – chronic graft-*versus*-host disease; ext – extensive; BM – bone marrow; PB – peripheral blood; haplo-HSCT – haploidentical hematopoietic stem cell transplantation; sAML – secondary acute myeloid leukemia


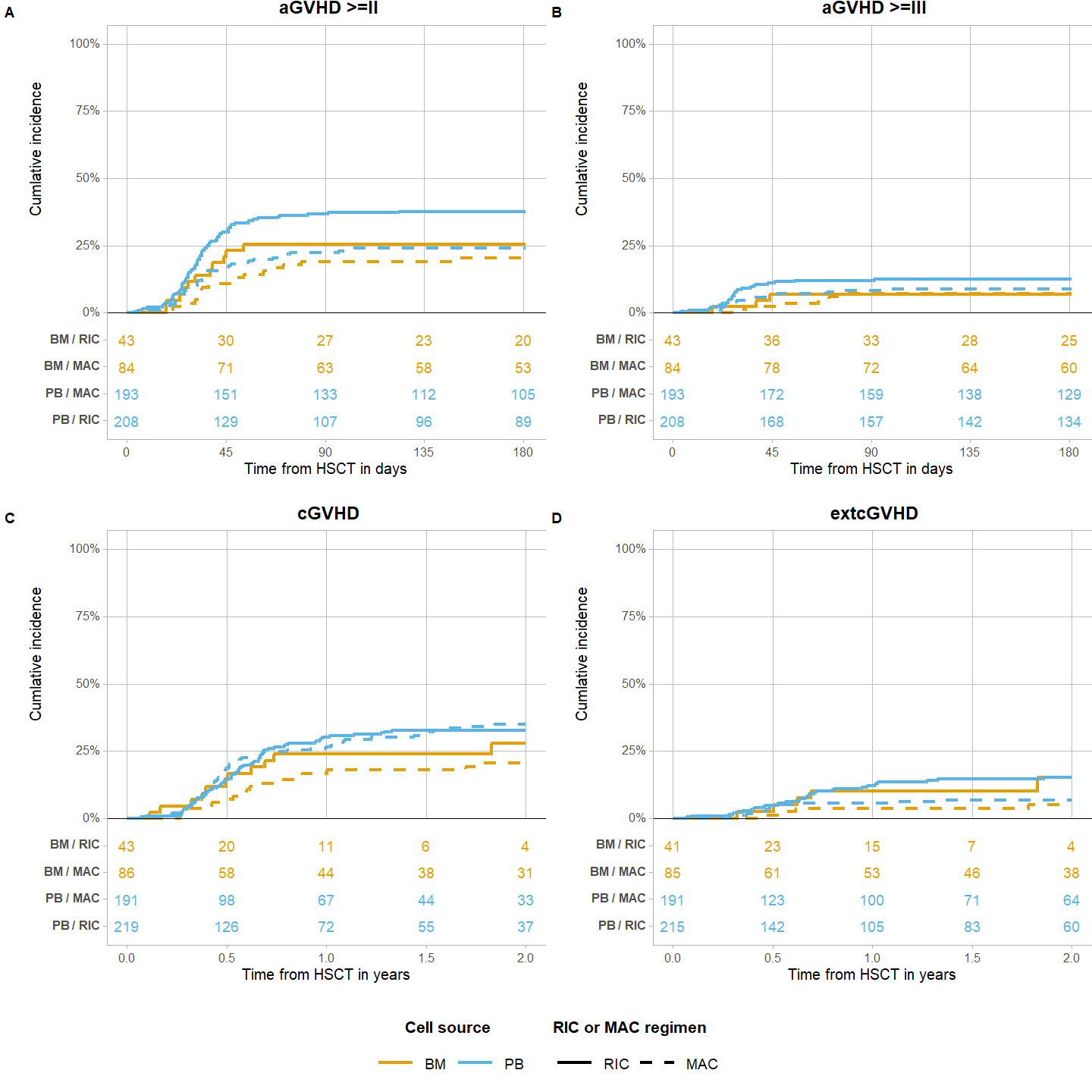

Supplement: Supplementary file 2 — Supplementary Figures S1-S2 [file 41409_2026_2823_MOESM2_ESM.docx]
