# Supplementary material for: Peripheral blood stem cells versus bone marrow graft for non-T-depleted haploidentical transplantation with post-transplant cyclophosphamide in patients with secondary acute myeloid leukemia in first complete remission: A study from the ALWP/EBMT
Source: Bone Marrow Transplant. 2026 Mar 25;61(5):559–68. doi: 10.1038/s41409-026-02823-2 (PMC13152805; doi:10.1038/s41409-026-02823-2)
Supplement: Supplementary file 3 — Supplementary Tables S1-S5 [file 41409_2026_2823_MOESM3_ESM.docx]

**Supplementary Table S1. Conditioning regimens**

| **Variable** | **Overall Population**  N = 554 | **BM**  N = 136 | **PBSC**  N = 418 |  |
| --- | --- | --- | --- | --- |
| TBI-based | 108 (19.5%) | 25 (18.4%) | 83 (19.9%) |  |
| TBF-based | 252 (45.6%) | 94 (69.1%) | 158 (37.9%) |  |
| TreoFlu-based | 50 (9.0%) | 6 (4.4%) | 44 (10.6%) |  |
| BuFlu-based | 100 (18.1%) | 8 (5.9%) | 92 (22.1%) |  |
| BuCy-based | 5 (0.9%) | 0 (0.0%) | 5 (1.2%) |  |
| FluMel-based | 35 (6.3%) | 3 (2.2%) | 32 (7.7%) |  |
| Other | 3 (0.5%) | 0 (0.0%) | 3 (0.7%) |  |
| Missing | 1 | 0 | 1 |  |

Abbreviations: BM-bone marrow; PBSC- peripheral blood stem cells; TBI-total body irradiation; Mel-melphalan; Bu-busulfan; Flu-fludarabine; Cy-cytoxan; TBF-thiotepa, Bu and Flu; Treo-Treosulfan

Results are expressed as frequency (%)

**Supplementary Table S2. Anti-GVHD prophylaxis regimens**

| **Variable** | **Overall population**  N = 554 | **BM**  N = 136 | **PBSC**  N = 418 | **p-value** |
| --- | --- | --- | --- | --- |
|  |  |  |  | 0.014 |
| CSA-based | 14 (2.7%) | 8 (6.4%) | 6 (1.5%) |  |
| MMF+CSA-based | 300 (56.9%) | 75 (60.0%) | 225 (56.0%) |  |
| MMF+CSA+TACRO-based | 9 (1.7%) | 0 (0.0%) | 9 (2.2%) |  |
| MMF+SIRO-based | 26 (4.9%) | 2 (1.6%) | 24 (6.0%) |  |
| MMF+TACRO-based | 155 (29.4%) | 34 (27.2%) | 121 (30.1%) |  |
| TACRO-based | 20 (3.8%) | 5 (4.0%) | 15 (3.7%) |  |
| Other | 3 (0.6%) | 1 (0.8%) | 2 (0.5%) |  |
| Missing | 27 | 11 | 16 |  |

Abbreviations: BM-bone marrow; PBSC- peripheral blood stem cells; CSA- cyclosporine A; MMF- mycophenolate mofetil; SIRO- sirolimus; TACRO- tacrolimus

Results are expressed as frequency (%)

**Supplementary Table S3. Estimation of outcomes according to conditioning intensity: reduced intensity conditioning/myeloablative condition (RIC/MAC): A – main transplantation outcomes, B – acute and chronic graft versus host diseases**

**A.**

| **Estimate (95% CI)** | **OS (2y)** | **LFS (2y)** | **GRFS (2y)** | **RI (2y)** | **NRM (2y)** |
| --- | --- | --- | --- | --- | --- |
| **Overall** | 59.2 (54.7 - 63.3) | 52.9 (48.5 - 57.1) | 41.5 (37.2 - 45.8) | 22.1 (18.6 - 25.8) | 25 (21.4 - 28.8) |
| **BM / RIC** | 38.6 (23.2 - 53.7) | 38.6 (23.7 - 53.4) | 22.1 (9.6 - 37.8) | 36.6 (21.6 - 51.7) | 24.8 (13.2 - 38.2) |
| **BM / MAC** | 69.8 (58.8 - 78.4) | 62.5 (51.2 - 71.9) | 56.2 (44.8 - 66.1) | 16.9 (9.7 - 25.8) | 20.6 (12.8 - 29.7) |
| **PBSC / MAC** | 62.1 (54.5 - 68.7) | 54.9 (47.3 - 61.8) | 45.6 (38.2 - 52.8) | 19.9 (14.4 - 26) | 25.3 (19.3 - 31.7) |
| **PBSC / RIC** | 56.4 (49.2 - 62.9) | 50.2 (43.1 - 56.8) | 35.5 (29 - 42.1) | 23.3 (17.7 - 29.3) | 26.5 (20.7 - 32.6) |

**B.**

| **Estimate (95% CI)** | **aGVHD >=II (180d)** | **aGVHD >=III (180d)** | **cGVHD (2y)** | **ext cGVHD (2y)** |
| --- | --- | --- | --- | --- |
| **Overall** | 28.8 (25 - 32.7) | 9.8 (7.5 - 12.6) | 31.1 (27.1 - 35.2) | 10.4 (7.9 - 13.3) |
| **BM / RIC** | 24.4 (13 - 37.8) | 6.7 (1.7 - 16.5) | 28.9 (15.4 - 43.9) | 14.1 (4.5 - 28.9) |
| **BM / MAC** | 20.3 (12.4 - 29.5) | 7.1 (2.9 - 14) | 20.7 (12.6 - 30.1) | 5.1 (1.6 - 11.7) |
| **PBSC / MAC** | 23.9 (18.1 - 30.2) | 8.8 (5.4 - 13.4) | 35 (27.9 - 42.1) | 6.7 (3.7 - 11.1) |
| **PBSC / RIC** | 37.7 (31.1 - 44.2) | 12.5 (8.4 - 17.4) | 32.8 (26.5 - 39.2) | 15.1 (10.6 - 20.4) |

Abbreviations: RIC-reduced intensity conditioning; MAC-myeloablative conditioning; OS-overall survival; LFS-leukemia-free survival; GRFS-GVHD-free, relapse-free survival; RI-relapse incidence; NRM-non-relapse mortality; GVHD- graft-*versus*-host disease; aGVHD-acute GVHD; cGVHD-chronic GVHD; ext cGVHD-extensive chronic GVHD; y-year; d- day; BM-bone marrow; PBSC - peripheral blood stem cells; results are expressed as frequency (%)

**Supplementary Table S4. Multivariate analysis for reduced intensity conditioning: A – main transplant outcomes, B – graft versus host diseases**

**A**

|  | **OS** | | **LFS** | | **GRFS** | | **RI** | | **NRM** | |
| --- | --- | --- | --- | --- | --- | --- | --- | --- | --- | --- |
| **Variable** | **HR (95%CI)** | **P-value** | **HR (95%CI)** | **P-value** | **HR (95%CI)** | **P-value** | **HR (95%CI)** | **P-value** | **HR (95%CI)** | **p-value** |
| **Cell source: BM vs PBSC** | 0.78 (0.44-1.38) | 0.392 | 0.88 (0.5-1.54) | 0.65 | 0.96 (0.58-1.59) | 0.871 | 1.01 (0.45-2.23) | 0.988 | 0.87 (0.33-2.33) | 0.786 |
| **Karnofsky score:**  **<90 vs >= 90** | 0.6 (0.39-0.92) | 0.019 | 0.61 (0.4-0.92) | 0.018 | 0.66 (0.45-0.95) | 0.026 | 0.73 (0.4-1.34) | 0.311 | 0.44 (0.22-0.88) | 0.019 |
| **Age of the patient at HSCT (per 10 years)** | 0.97 (0.78-1.2) | 0.759 | 0.91 (0.75-1.1) | 0.329 | 0.98 (0.81-1.17) | 0.783 | 0.85 (0.66-1.09) | 0.19 | 1.1 (0.74-1.64) | 0.628 |
| **Year of transplantation (per 5 years)** | 0.73 (0.45-1.17) | 0.189 | 0.78 (0.5-1.22) | 0.276 | 0.86 (0.58-1.29) | 0.464 | 0.53 (0.29-0.96) | 0.034 | 1.31 (0.57-3.03) | 0.527 |
| **Female donor to male patient: No vs Yes** | 1 (0.61-1.65) | 0.987 | 0.95 (0.59-1.52) | 0.822 | 0.97 (0.64-1.45) | 0.867 | 1.07 (0.56-2.03) | 0.835 | 0.85 (0.39-1.85) | 0.684 |
| **Cytogenetic AML classification: Favorable/Int/Missing vs Adverse** | 1.49 (0.97-2.27) | 0.068 | 1.47 (0.98-2.22) | 0.062 | 1.57 (1.09-2.26) | 0.015 | 2.04 (1.17-3.56) | 0.012 | 1.15 (0.57-2.33) | 0.695 |
| **Months between diagnosis and HSCT** | 1.02 (0.94-1.1) | 0.663 | 1 (0.93-1.08) | 0.992 | 1 (0.94-1.07) | 0.945 | 1 (0.89-1.11) | 0.938 | 1.01 (0.9-1.15) | 0.83 |
| **Age of the donor at HSCT (per 10 years)** | 1.04 (0.87-1.25) | 0.651 | 0.98 (0.82-1.16) | 0.808 | 0.95 (0.81-1.1) | 0.46 | 0.84 (0.64-1.1) | 0.208 | 0.97 (0.75-1.26) | 0.814 |
| **HCT-CI: 0 vs >=3** | 1.5 (0.93-2.41) | 0.098 | 1.5 (0.95-2.37) | 0.082 | 1.15 (0.77-1.71) | 0.504 | 1.96 (1.02-3.76) | 0.043 | 1.29 (0.6-2.79) | 0.514 |
| **HCT-CI: 0 vs 1-2** | 1.1 (0.6-2.01) | 0.756 | 1.1 (0.62-1.94) | 0.744 | 0.81 (0.49-1.32) | 0.393 | 1.01 (0.43-2.35) | 0.987 | 1.03 (0.42-2.5) | 0.954 |

**B.**

|  | **aGVHD >=II** | | **aGVHD >=III** | | **cGVHD** | | **ext cGVHD** | |
| --- | --- | --- | --- | --- | --- | --- | --- | --- |
| **Variable** | **HR (95%CI)** | **P-value** | **HR (95%CI)** | **P-value** | **HR (95%CI)** | **P-value** | **HR (95%CI)** | **p-value** |
| **Cell source: BM vs PBSC** | 1.47 (0.65-3.29) | 0.352 | 4.98 (0.59-42.36) | 0.141 | 0.94 (0.42-2.11) | 0.887 | 1.52 (0.32-7.37) | 0.601 |
| **Karnofsky score:**  **<90 vs >= 90** | 0.75 (0.44-1.28) | 0.293 | 1.06 (0.35-3.18) | 0.92 | 1.83 (0.99-3.39) | 0.054 | 1.07 (0.42-2.73) | 0.882 |
| **Age of the patient at HSCT (per 10 years)** | 1.19 (0.86-1.64) | 0.287 | 1.24 (0.59-2.63) | 0.566 | 1.74 (1.2-2.52) | 0.003 | 1.29 (0.78-2.14) | 0.317 |
| **Year of transplantation (per 5 years)** | 1.49 (0.76-2.93) | 0.242 | 1 (0.27-3.66) | 0.999 | 0.89 (0.46-1.7) | 0.716 | 0.85 (0.32-2.3) | 0.752 |
| **Female donor to male patient: No vs Yes** | 0.53 (0.28-1.02) | 0.058 | 0.47 (0.12-1.78) | 0.263 | 1.4 (0.78-2.51) | 0.259 | 1.15 (0.45-2.94) | 0.766 |
| **Cytogenetic AML classification: Favorable/Int/Missing vs Adverse** | 0.94 (0.55-1.61) | 0.819 | 0.82 (0.29-2.34) | 0.707 | 2.1 (1.21-3.66) | 0.009 | 3.09 (1.32-7.25) | 0.01 |
| **Months between diagnosis and HSCT** | 0.96 (0.87-1.06) | 0.408 | 1 (0.83-1.2) | 0.982 | 0.98 (0.88-1.09) | 0.688 | 1.06 (0.91-1.23) | 0.449 |
| **Age of the donor at HSCT (per 10 years)** | 1.04 (0.84-1.31) | 0.701 | 1.01 (0.66-1.53) | 0.971 | 0.96 (0.75-1.22) | 0.711 | 0.96 (0.66-1.41) | 0.846 |
| **HCT-CI: 0 vs >=3** | 0.94 (0.53-1.66) | 0.825 | 0.8 (0.26-2.47) | 0.699 | 1.25 (0.67-2.34) | 0.476 | 0.79 (0.29-2.18) | 0.651 |
| **HCT-CI: 0 vs 1-2** | 0.88 (0.45-1.73) | 0.708 | 0.49 (0.13-1.95) | 0.315 | 1.24 (0.62-2.51) | 0.543 | 1.16 (0.38-3.58) | 0.792 |

Abbreviations: OS-overall survival; LFS-leukemia-free survival; GRFS-GVHD-free, relapse-free survival; RI-relapse incidence; NRM-non-relapse mortality; GVHD- graft-*versus*-host disease; aGVHD-acute GVHD; cGVHD-chronic GVHD; Ext cGVHD-extensive chronic GVHD; BM-bone marrow; PBSC - peripheral blood stem cells; int – intermediate; HR-hazard ratio; CI-confidence interval; BM-bone marrow; HSCT-hematopoietic stem cell transplantation; AML-acute myeloid leukemia; HCT-CI-hematopoietic cell transplantation–specific comorbidity index

**Supplementary Table S5. Multivariate analysis for myeloablative conditioning: A – main transplantation outcomes, B – acute and chronic graft versus host diseases**

**A**

|  | **OS** | | **LFS** | | **GRFS** | | **RI** | | **NRM** | |
| --- | --- | --- | --- | --- | --- | --- | --- | --- | --- | --- |
| **Variable** | **HR (95%CI)** | **P-value** | **HR (95%CI)** | **P-value** | **HR (95%CI)** | **P-value** | **HR (95%CI)** | **P-value** | **HR (95%CI)** | **P-value** |
| **Cell source: BM vs PBSC** | 0.78 (0.44-1.38) | 0.392 | 0.88 (0.5-1.54) | 0.65 | 0.96 (0.58-1.59) | 0.871 | 1.01 (0.45-2.23) | 0.988 | 0.87 (0.33-2.33) | 0.786 |
| **Karnofsky score:**  **<90 vs >= 90** | 0.6 (0.39-0.92) | 0.019 | 0.61 (0.4-0.92) | 0.018 | 0.66 (0.45-0.95) | 0.026 | 0.73 (0.4-1.34) | 0.311 | 0.44 (0.22-0.88) | 0.019 |
| **Age of the patient at HSCT (per 10 years)** | 0.97 (0.78-1.2) | 0.759 | 0.91 (0.75-1.1) | 0.329 | 0.98 (0.81-1.17) | 0.783 | 0.85 (0.66-1.09) | 0.19 | 1.1 (0.74-1.64) | 0.628 |
| **Year of transplantation (per 5 years)** | 0.73 (0.45-1.17) | 0.189 | 0.78 (0.5-1.22) | 0.276 | 0.86 (0.58-1.29) | 0.464 | 0.53 (0.29-0.96) | 0.034 | 1.31 (0.57-3.03) | 0.527 |
| **Female donor to male patient: No vs Yes** | 1 (0.61-1.65) | 0.987 | 0.95 (0.59-1.52) | 0.822 | 0.97 (0.64-1.45) | 0.867 | 1.07 (0.56-2.03) | 0.835 | 0.85 (0.39-1.85) | 0.684 |
| **Cytogenetic AML classification: Favorable/Int/Missing vs Adverse** | 1.49 (0.97-2.27) | 0.068 | 1.47 (0.98-2.22) | 0.062 | 1.57 (1.09-2.26) | 0.015 | 2.04 (1.17-3.56) | 0.012 | 1.15 (0.57-2.33) | 0.695 |
| **Months between diagnosis and HSCT** | 1.02 (0.94-1.1) | 0.663 | 1 (0.93-1.08) | 0.992 | 1 (0.94-1.07) | 0.945 | 1 (0.89-1.11) | 0.938 | 1.01 (0.9-1.15) | 0.83 |
| **Age of the donor at HSCT (per 10 years)** | 1.04 (0.87-1.25) | 0.651 | 0.98 (0.82-1.16) | 0.808 | 0.95 (0.81-1.1) | 0.46 | 0.84 (0.64-1.1) | 0.208 | 0.97 (0.75-1.26) | 0.814 |
| **HCT-CI: 0 vs >=3** | 1.5 (0.93-2.41) | 0.098 | 1.5 (0.95-2.37) | 0.082 | 1.15 (0.77-1.71) | 0.504 | 1.96 (1.02-3.76) | 0.043 | 1.29 (0.6-2.79) | 0.514 |
| **HCT-CI: 0 vs 1-2** | 1.1 (0.6-2.01) | 0.756 | 1.1 (0.62-1.94) | 0.744 | 0.81 (0.49-1.32) | 0.393 | 1.01 (0.43-2.35) | 0.987 | 1.03 (0.42-2.5) | 0.954 |

**B.**

|  | **aGVHD >=II** | | **aGVHD >=III** | | **cGVHD** | | **extcGVHD** | |
| --- | --- | --- | --- | --- | --- | --- | --- | --- |
| **Variable** | **HR (95%CI)** | **P-value** | **HR (95%CI)** | **P-value** | **HR (95%CI)** | **P-value** | **HR (95%CI)** | **P-value** |
| **Cell source: BM vs PBSC** | 1.47 (0.65-3.29) | 0.352 | 4.98 (0.59-42.36) | 0.141 | 0.94 (0.42-2.11) | 0.887 | 1.52 (0.32-7.37) | 0.601 |
| **Karnofsky score:**  **<90 vs >= 90** | 0.75 (0.44-1.28) | 0.293 | 1.06 (0.35-3.18) | 0.92 | 1.83 (0.99-3.39) | 0.054 | 1.07 (0.42-2.73) | 0.882 |
| **Age of the patient at HSCT (per 10 years)** | 1.19 (0.86-1.64) | 0.287 | 1.24 (0.59-2.63) | 0.566 | 1.74 (1.2-2.52) | 0.003 | 1.29 (0.78-2.14) | 0.317 |
| **Year of transplantation (per 5 years)** | 1.49 (0.76-2.93) | 0.242 | 1 (0.27-3.66) | 0.999 | 0.89 (0.46-1.7) | 0.716 | 0.85 (0.32-2.3) | 0.752 |
| **Female donor to male patient: No vs Yes** | 0.53 (0.28-1.02) | 0.058 | 0.47 (0.12-1.78) | 0.263 | 1.4 (0.78-2.51) | 0.259 | 1.15 (0.45-2.94) | 0.766 |
| **Cytogenetic AML classification: Favorable/Int/Missing vs Adverse** | 0.94 (0.55-1.61) | 0.819 | 0.82 (0.29-2.34) | 0.707 | 2.1 (1.21-3.66) | 0.009 | 3.09 (1.32-7.25) | 0.01 |
| **Months between diagnosis and HSCT** | 0.96 (0.87-1.06) | 0.408 | 1 (0.83-1.2) | 0.982 | 0.98 (0.88-1.09) | 0.688 | 1.06 (0.91-1.23) | 0.449 |
| **Age of the donor at HSCT (per 10 years)** | 1.04 (0.84-1.31) | 0.701 | 1.01 (0.66-1.53) | 0.971 | 0.96 (0.75-1.22) | 0.711 | 0.96 (0.66-1.41) | 0.846 |
| **HCT-CI: 0 vs >=3** | 0.94 (0.53-1.66) | 0.825 | 0.8 (0.26-2.47) | 0.699 | 1.25 (0.67-2.34) | 0.476 | 0.79 (0.29-2.18) | 0.651 |
| **HCT-CI: 0 vs 1-2** | 0.88 (0.45-1.73) | 0.708 | 0.49 (0.13-1.95) | 0.315 | 1.24 (0.62-2.51) | 0.543 | 1.16 (0.38-3.58) | 0.792 |

Abbreviations: OS-overall survival; LFS-leukemia-free survival; GRFS-GVHD-free, relapse-free survival; RI-relapse incidence; NRM-non-relapse mortality; GVHD- graft-*versus*-host disease; aGVHD-acute GVHD; cGVHD-chronic GVHD; Ext cGVHD-extensive chronic GVHD; BM-bone marrow; PBSC - peripheral blood stem cells; int – intermediate; HR-hazard ratio; CI-confidence interval; BM-bone marrow; HSCT-hematopoietic stem cell transplantation; AML-acute myeloid leukemia; HCT-CI-hematopoietic cell transplantation–specific comorbidity index
